# Effects of Phytochemical P-Glycoprotein Modulators on the Pharmacokinetics and Tissue Distribution of Doxorubicin in Mice

**DOI:** 10.3390/molecules23020349

**Published:** 2018-02-07

**Authors:** Tae Hwan Kim, Soyoung Shin, Sun Dong Yoo, Beom Soo Shin

**Affiliations:** 1 College of Pharmacy, Catholic University of Daegu, Gyeongsan 38430, Gyeongbuk, Korea; thwnkim86@gmail.com; 2 College of Pharmacy, Wonkwang University, Iksan 54538, Jeonbuk, Korea; shins@wku.ac.kr; 3 School of Pharmacy, Sungkyunkwan University, Suwon 16419, Gyeonggi-do, Korea; sdyoo@skku.ac.kr

**Keywords:** P-glycoprotein, doxorubicin, pharmacokinetics, piperine, capsaicin, [6]-gingerol

## Abstract

Pungent spice constituents such as piperine, capsaicin and [6]-gingerol consumed via daily diet or traditional Chinese medicine, have been reported to possess various pharmacological activities. These dietary phytochemicals have also been reported to inhibit P-glycoprotein (P-gp) in vitro and act as an alternative to synthetic P-gp modulators. However, the in vivo effects on P-gp inhibition are currently unknown. This study aimed to test the hypothesis that phytochemical P-gp inhibitors, i.e., piperine, capsaicin and [6]-gingerol, modulate the in vivo tissue distribution of doxorubicin, a representative P-gp substrate. Mice were divided into four groups and each group was pretreated with intraperitoneal injections of control vehicle, piperine, capsaicin, or [6]-gingerol and doxorubicin (1 mg/kg) was administered via the penile vein. The concentrations of the phytochemicals and doxorubicin in the plasma and tissues were determined by LC-MS/MS. The overall plasma concentration-time profiles of doxorubicin were not significantly affected by piperine, capsaicin, or [6]-gingerol. In contrast, doxorubicin accumulation was observed in tissues pretreated with piperine or capsaicin. The tissue to plasma partition coefficients, K_p_, for the liver and kidney were higher in the piperine-pretreated group, while the K_p_ for kidney, brain and liver were higher in the capsaicin-pretreated group. [6]-Gingerol did not affect doxorubicin tissue distribution. The data demonstrated that the phytochemicals modulated doxorubicin tissue distribution, which suggested their potential to induce food-drug interactions and act as a strategy for the delivery of P-gp substrate drugs to target tissues and tumors.

## 1. Introduction

Pungent phytochemicals are consumed worldwide every day. For example, the average amount of piperine from black pepper consumed in the U.S. was reported to be 21.0 mg/person/day [1]. The maximum daily intake of capsaicin in the U.S. and Europe from mild chilies and paprika was estimated to be 1.5 mg/person/day [2,3]. These spicy phytochemicals have been reported to possess various pharmacological activities. They are also known to inhibit P-glycoprotein (P-gp) in vitro [4,5,6,7,8] and the potential use of these phytochemicals as an alternative to synthetic P-gp inhibitors for the reversal of multidrug resistance (MDR) in chemotherapy has been suggested [7,9].

P-gp is an ATP-dependent drug efflux transporter for pumping out xenobiotic compounds to outside of the cell. P-gp plays a central role in drug absorption and disposition in vivo and is a critical determinant in the pharmacokinetics and clinical response of many drugs. For example, the presence of P-gp strongly reduced the brain accumulation of many drugs and the penetration of substrates into the brain was increased by up to 46-fold in P-gp knockout mice [10,11,12]. Substantial accumulation of digoxin in various tissues including brain, liver and kidney has been reported in mdr1a (−/−) mice [13]. It has also been reported that P-gp in the epithelium of the gut limits oral bioavailability of paclitaxel by a direct secretion of paclitaxel into the intestinal lumen [14]. Furthermore, the presence of P-gp in tumor cells was reported as a major contributory mechanism of MDR in tumors, which is a leading cause of chemotherapy failure [15,16].

Therefore, P-gp modulation can exert dramatic effects on the systemic disposition of the substrate drugs including anticancer agents and ultimately benefit clinical efficacy. Several synthetic modulators, such as verapamil, quinidine, talinolol, clarithromycin, erythromycin, itraconazole, ritonavir and elacridar, have been found to inhibit P-gp and mediate clinically relevant drug-drug interactions [17]. Nevertheless, these synthetic P-gp modulators may have potential undesirable effects owing to their nonspecificity to P-gp [18,19]. Moreover, relatively high doses of P-gp modulators are usually required, which may be associated with unwanted drug-drug interactions and potential toxicity [9,19,20,21].

Piperine (from black pepper, Piper nigrum), capsaicin (from chili pepper, *Capsicum annuum*) and [6]-gingerol (from ginger, *Zingiber officinale*) are bioactive components of pungent spices which are commonly consumed in daily diet. In addition to their culinary uses, they have been used in traditional Chinese medicine; various pharmacological activities have been demonstrated, including anti-inflammatory [22,23,24,25,26], anti-oxidative [22,25,27], analgesic [26] and cancer preventive [28,29] effects. The modulatory effects of these phytochemicals on P-gp have also been indicated in vitro (Table 1). For example, piperine inhibited the transport of P-gp substrates, digoxin and cyclosporine A, in Caco-2 cells with IC50 values of 15.5 and 74.1 μmol/L, respectively [4]. The accumulation of rhodamine 123 increased by 4-fold in parallel to the decrease of efflux in the presence of 50 μmol/L capsaicin in human multidrug-resistant carcinoma KB-C2 cells [7]. Capsaicin also inhibited digoxin efflux mediated by P-gp in Caco-2 cells, with no resulting cytotoxicity [6]. A major constituent of ginger, [6]-gingerol, inhibited P-gp-mediated digoxin transport in L-MDR1 and Caco-2 cells [8]. In the presence of [6]-gingerol, the accumulation of rhodamine 123 increased by 2.24-fold in KB-C2 cells [7].

These natural compounds may be less toxic than the available synthetic inhibitors and therefore have the potential to be used as P-gp modulators [9]. However, it remains to be verified whether these P-gp modulating phytochemicals could affect the tissue distribution of substrate drugs in vivo and to confirm their potential clinical applications. In the present study, we therefore aimed to evaluate the effects of the phytochemicals on the in vivo tissue distribution of P-gp substrate by using doxorubicin as a model P-gp substrate. Doxorubicin is a widely used chemotherapeutic agent that is known to be a selective P-gp substrate and could induce expression of P-gp in tumor cells [31,32]. The changes in the pharmacokinetics of doxorubicin and the tissue distribution were studied by the concurrent administration of P-gp modulating phytochemicals, i.e., piperine, capsaicin and [6]-gingerol, in mice.

## 2. Results

### 2.1. Pharmacokinetics of Piperine, Capsaicin and [6]-Gingerol

**Figure 1:**
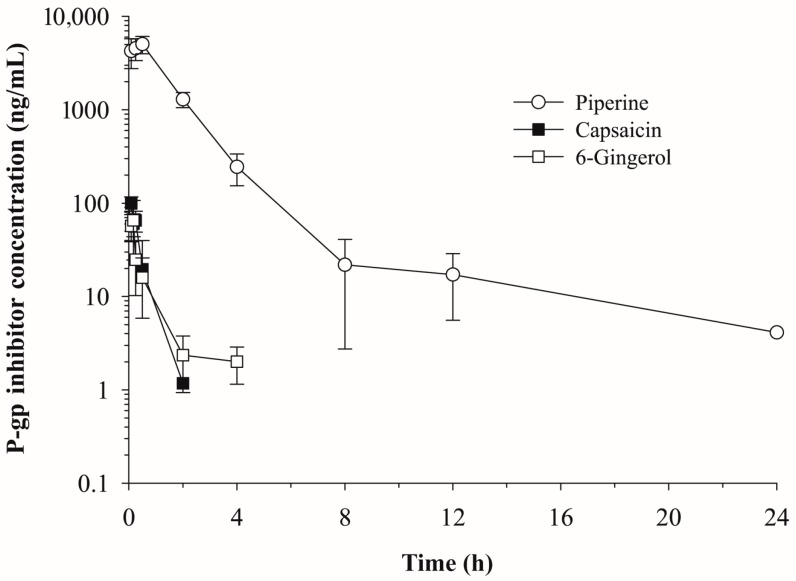
Plasma concentration vs. time profiles of piperine, capsaicin and [6]-gingerol after intraperitoneal injection in mice (*n* = 6, mean ± SD).

The plasma concentration vs. time profiles of the phytochemicals, piperine (10 mg/kg), capsaicin (5 mg/kg) and [6]-gingerol (5 mg/kg), after intraperitoneal administration are shown in Figure 1. The non-compartmental pharmacokinetic parameters of each phytochemical are summarized in Table 2. Although the piperine plasma concentration declined with an elimination half-life (t_1/2_) of 6.30 h, capsaicin and [6]-gingerol were eliminated rapidly from plasma with t_1/2_ values of 0.33 h and 1.36 h, respectively and were not detected 2–4 h after administration. A higher maximum plasma concentration (C_max_), higher area under the curve (AUC_inf_), smaller systemic clearance (CL/F) and smaller volume of distribution (V_z_/F) were also observed for piperine compared with those for capsaicin and [6]-gingerol (Table 2).

**Figure 2:**
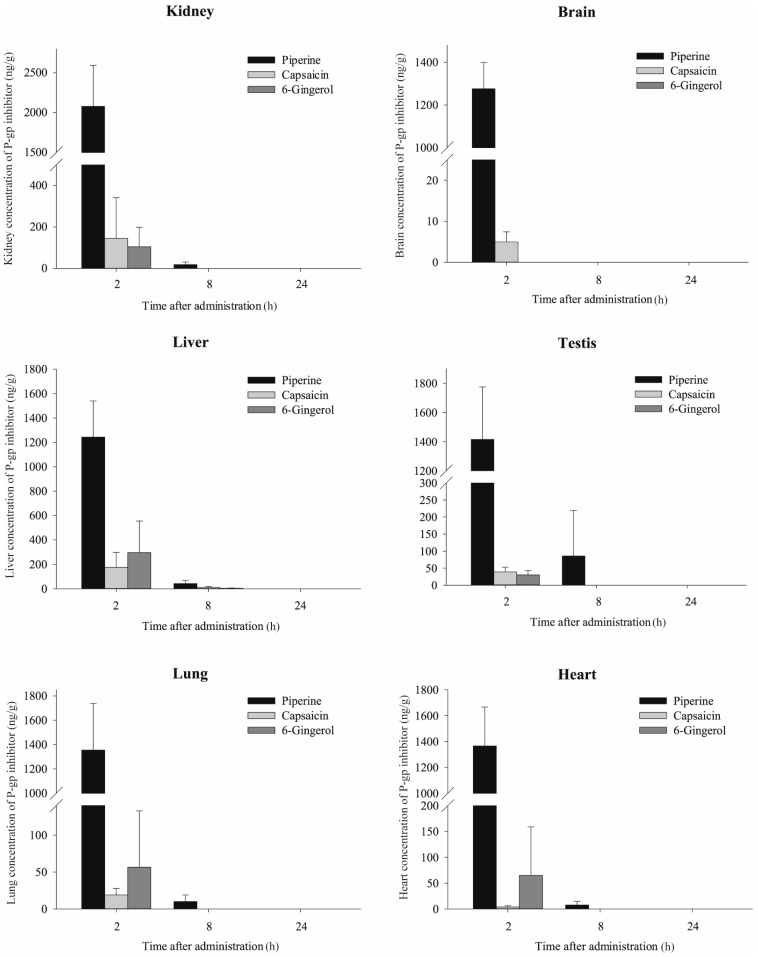
Tissue concentrations of piperine, capsaicin and [6]-gingerol after intraperitoneal administration in mice (*n* = 6, mean ± SD).

Consistent with their plasma concentration vs. time profiles, capsaicin and [6]-gingerol were not detected in the most tissues except in liver at 8 h after administration, whereas piperine was distributed into various tissues 8 h after administration (Figure 2). In most tissues, the concentration of piperine was much higher than that of capsaicin and [6]-gingerol. None of the phytochemicals was detected in the tissues 24 h after administration. Piperine concentrations were found to be the highest in the kidney, with a tissue to plasma partition coefficient, K_p_, kidney = 1.69 ± 0.28, at 2 h after administration, followed by the testis, heart, lung, brain and liver. Capsaicin was distributed most extensively in the liver, followed by the testis, kidney, lung, brain and heart at 2 h after administration (Figure 2). The tissue distribution of [6]-gingerol was most pronounced in the liver, followed by the kidney and lung but was not detected in the brain, heart and testis 2 h after administration (Figure 2).

### 2.2. Effects of Piperine, Capsaicin and [6]-Gingerol on the Plasma Pharmacokinetics of Doxorubicin

**Figure 3:**
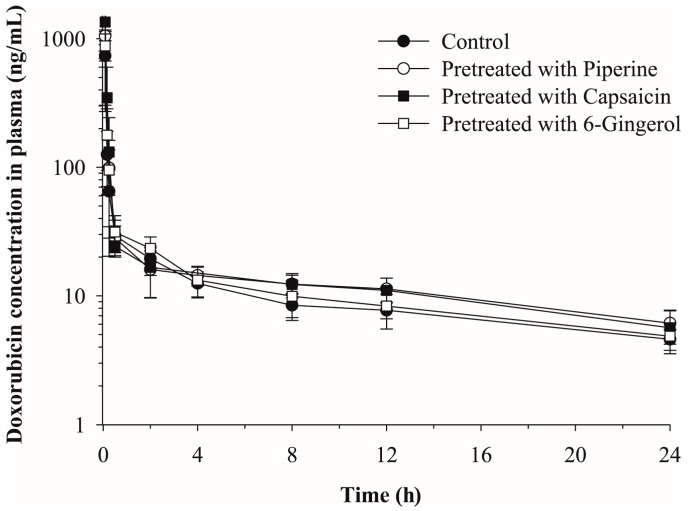
Plasma concentration vs. time profiles of doxorubicin after intravenous injection of doxorubicin at 1 mg/kg in mice pretreated with vehicle (control), piperine, capsaicin, or [6]-gingerol (*n* = 6, mean ± SD).

The plasma concentration vs. time profiles of doxorubicin after intravenous administration in mice that were pretreated with a phytochemical P-gp modulator, i.e., piperine, capsaicin, or [6]-gingerol, or vehicle control, are shown in Figure 3. The non-compartmental pharmacokinetic parameters of doxorubicin are summarized in Table 3. Owing to the disruptive sampling methodology, the pharmacokinetic parameters were estimated by using the mean values and the standard deviations of each parameter could not be obtained. As shown in Figure 3, the overall pharmacokinetic profile of doxorubicin was not significantly affected by the phytochemicals. No significant changes in the t_1/2_ of doxorubicin were observed among groups (Table 3). A higher initial plasma concentration (C_0_) and greater AUC_inf_ values of doxorubicin were observed in the capsaicin-pretreated group compared to control group. However, any statistical analyses could not be performed due to the disruptive sampling (Table 3).

### 2.3. Effects of Piperine, Capsaicin and [6]-Gingerol on Tissue Distribution of Doxorubicin

**Figure 4:**
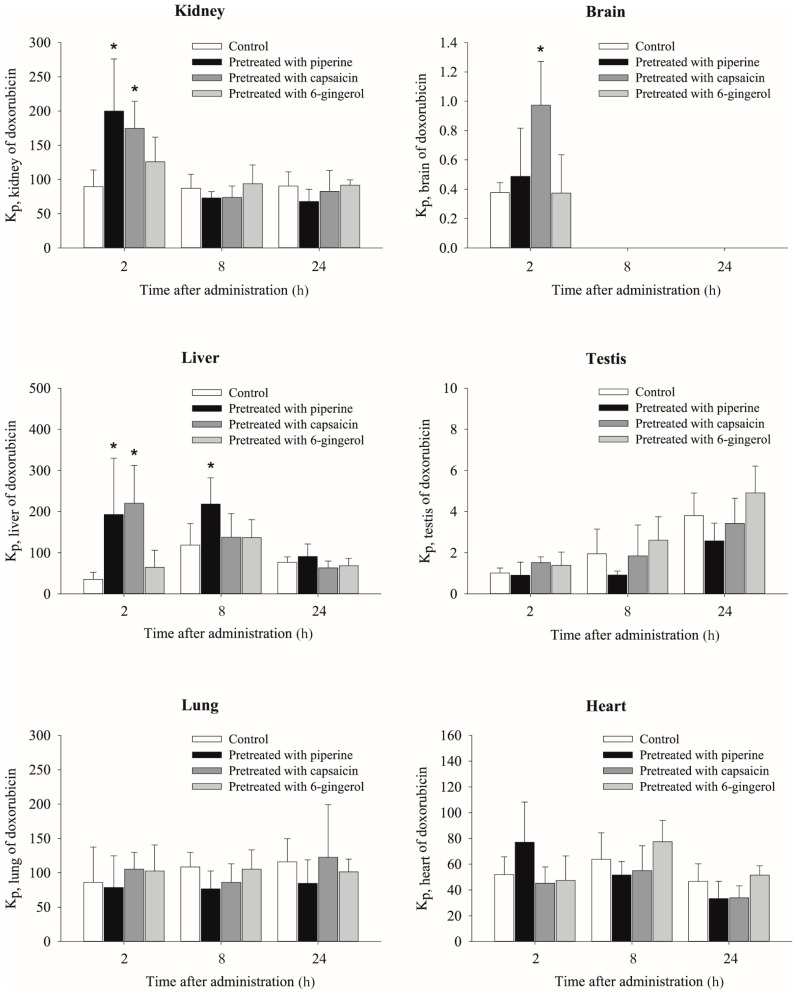
Tissue to plasma partition coefficients (K_p_) of doxorubicin 2, 8 and 24 h after intravenous injection of doxorubicin at 1 mg/kg in mice pretreated with vehicle (control), piperine, capsaicin, or [6]-gingerol (*n* = 6, mean ± SD). * *p* < 0.05 vs. control.

Doxorubicin concentrations in the plasma, kidney, brain, liver, testis, lung and heart at 2, 8 and 24 h after intravenous injection in mice pretreated with piperine, capsaicin and [6]-gingerol are presented in Table 4. The tissue to plasma partition coefficients (K_p_) of doxorubicin in the six different tissues are shown in Figure 4. Overall plasma concentrations of doxorubicin were all similar among groups. Doxorubicin was extensively distributed into the kidney, lung, heart and liver with K_p_ values of >35.4, whereas distribution into the testis and brain was lower.

As shown in Figure 4, the K_p_ values of doxorubicin for several tissues were increased in piperine- and capsaicin-pretreated mice compared with those in control mice but [6]-gingerol did not significantly affect the K_p_ of doxorubicin. Two hours after administration, the K_p_ of doxorubicin in the kidney was significantly increased by pretreatment with piperine (2.23-fold) and capsaicin (1.95-fold) comparing with control. Pretreatment with capsaicin also significantly increased the K_p_ of doxorubicin at 2 h in the brain (3.33-fold). The K_p_ of doxorubicin in the liver was largely increased by piperine and capsaicin pretreatment by 5.44- and 6.21-fold, respectively. The increase in K_p_ for the liver after piperine pretreatment was maintained for 8 h after doxorubicin administration. The K_p_ values of doxorubicin in the testis, lung and heart were not significantly different among all the groups.

## 3. Discussion

In this study, the modulatory effects of the phytochemicals on P-gp were evaluated in vivo by using doxorubicin as a model P-gp substrate. Although alteration in oral bioavailabilities of various drugs by combination with the pungent phytochemicals have been well demonstrated [33,34,35,36,37,38,39,40], no information is available regarding their effects on tissue distribution of P-gp substrates. Therefore, the tissue distribution of doxorubicin after intravenous injection were evaluated in mice pretreated with piperine, capsaicin, or [6]-gingerol.

The present results clearly demonstrated that piperine and capsaicin modulated the pharmacokinetics and tissue distribution of doxorubicin in vivo. Although the P-gp inhibitory activities of various dietary phytochemicals have been well established in vitro (Table 1), the present study provided direct experimental evidence to support their potential use as in vivo P-gp modulators. The different magnitude of the in vivo effects of the phytochemicals on the doxorubicin tissue distribution may be attributed to their different in vitro activities as well as in vivo pharmacokinetics. After intraperitoneal administration, the phytochemicals may undergo gastrointestinal and hepatic first-pass metabolism. Although the absorption of piperine is efficient [41], capsaicin and [6]-gingerol are likely to undergo significant first-pass metabolism. Compared with piperine, both capsaicin and [6]-gingerol showed a similar pharmacokinetic profile with a short elimination half-life but capsaicin resulted in a more pronounced alteration in doxorubicin tissue distribution than [6]-gingerol. This was consistent with previous in vitro results, which resulted in a higher accumulation of daunorubicin in conjunction with capsaicin than [6]-gingerol [7].

Pharmacokinetic studies have demonstrated that doxorubicin is extensively distributed into a wide range of tissues, including the liver, gut, heart, stomach, kidney, lung and spleen [42]. The primary elimination route of doxorubicin is biliary secretion; only a moderate amount of the drug undergoes urinary excretion [43,44,45]. In rats, approximately 30% of the initial dose appeared in the bile within 3 h as unchanged doxorubicin [43]. In bile duct-cannulated rats, 33–35% of the injected doxorubicin was excreted in bile and only 4–8% was eliminated via urine in bladder-cannulated rats [45].

Our data, based on the K_p_ values, indicated that among the tested phytochemicals, piperine and capsaicin significantly increased doxorubicin distribution into the liver and kidney. In particular, the brain distribution was also significantly increased in the capsaicin-pretreated mice comparing with control-pretreated mice (K_p_ = 1.0 ± 0.3 vs. 0.3 ± 0.1). These phytochemical effects were most pronounced at 2 h, while the effects were minimal 8 and 24 h after doxorubicin administration (Table 4 and Figure 4), which may be due to their rapid elimination. Capsaicin and [6]-gingerol were disappeared from plasma within 2–4 h (Figure 1) and were not present in the tissues from 8 h after doxorubicin administration (Figure 2). Piperine had longer half-life comparing with other two phytochemicals and were detected at 8 h in the tissues although the concentration at 8 h was significantly less than that at 2 h (Figure 2). P-gp is exclusively expressed on the biliary canalicular membrane of hepatocytes and on the apical surface of the bile duct in the liver [46]. In the kidney, P-gp is concentrated on the apical surface of epithelial cells of the proximal tubules [46]. Thus, the inhibition of P-gp by piperine or capsaicin may result in a reduction of the doxorubicin excretion into bile and urine, which leads to the accumulation of doxorubicin in these tissues. This is consistent with the significant accumulation of digoxin in the liver and kidney in mdr1a (−/−) mice [13]. P-gp is also highly expressed at the endothelial cells of the blood-brain barrier [47], where it limits the tissue distribution of the substrates. The inhibition of P-gp increased the distribution into brain tissue. The brain tissue distribution of doxorubicin was significantly enhanced by capsaicin pre-treatment (Table 4 and Figure 4), which was similar to results seen in mdr1a (−/−) mice [12].

Although the overall plasma pharmacokinetics of doxorubicin was not significantly affected by piperine or capsaicin, the terminal phase concentrations and AUC of doxorubicin were slightly increased by P-gp-modulating phytochemicals. The inhibition of P-gp by piperine and capsaicin may result in the reduced excretion of doxorubicin into bile and urine and the accumulation of doxorubicin in these tissues, which leads to the increased systemic exposure. These results are similar to previous studies with mdr1a (−/−) mice. In the absence of mdr1a P-gp, the biliary excretion of doxorubicin was significantly reduced but the change in the plasma concentration was minimal except slightly prolonged half-life [12,48].

However, there are reports that plasma concentrations were significantly affected in the presence of pungent phytochemicals after oral administration. These effects of phytochemicals might be contributed to their modulating effects on intestinal P-gp and metabolizing enzymes, or alteration of the membrane dynamics following oral administration [49]. For example, piperine co-administration increased plasma concentrations of phenytoin [33], propranolol [34] and theophylline [34] following oral administrations, which may be associated with inhibition of intestinal P-gp and/or metabolic enzymes. Capsaicin also increased oral bioavailability of cyclosporin, which is a substrate for both cytochrome P450 3A and P-gp, in rats [40]. Major difference of the present study with these reports could be found in that this study examined the effects of phytochemicals administered by intraperitoneal injection on tissue distribution of doxorubicin after intravenous injection, which could avoid confounding factors associated with complex absorption kinetics including intestinal P-gp. The present results, i.e., no significant changes in doxorubicin plasma pharmacokinetics by the phytochemicals are also in agreement with the literature. Plasma pharmacokinetics of another P-gp substrate, fexofenadine following intravenous injection was not altered by piperine [36]. In contrast, piperine co-administration significantly increased fexofenadine plasma concentrations and its bioavailability after oral administration which may be associated with their interaction in the intestinal P-gp [36].

Although P-gp expression at the blood-testis barrier [50] and in the heart [51] has been reported, only a minor effect of phytochemicals was observed in the testis, lung, or heart in the present study. The insignificant effects of phytochemicals on doxorubicin distribution in these organs might arise from the different P-gp expression levels, other transport systems, or less distribution of the phytochemicals into these organs. In particular, capsaicin concentration in the heart was lower than other organs (Figure 2), which may result in its minor effect on the cardiac distribution of doxorubicin. The most severe toxicities of doxorubicin and other anthracyclines are cardiomyopathy and congestive heart failure [52]. Although the accumulation of doxorubicin in the heart was not observed after administration of the phytochemicals in our experimental setting, it is important to monitor the potential cardiotoxicity of doxorubicin that occurs by other P-gp modulators. High mortality and cardiotoxicity were observed following the administration of doxorubicin in combination with several P-gp modulators, such as cyclosporine A, tamoxifen and verapamil; this was partially caused by an increase in doxorubicin accumulation in the heart [53,54,55]. An increase of doxorubicin distribution to the heart was also reported in mdr1a (−/−) mice [12]. Although P-gp expression level is low in the human heart, the increased systemic exposure of doxorubicin may also increase the cardiac concentration of doxorubicin [55].

This is the first in vivo study to demonstrate the modulation of P-gp substrate drug distribution into various tissues by pungent phytochemicals. This study demonstrated that dietary phytochemical P-gp modulators, such as piperine and capsaicin, were able to modulate doxorubicin pharmacokinetics and tissue distribution in vivo. Among the tested phytochemicals, capsaicin could serve as a promising agent to deliver P-gp substrates into target tissues or to reverse MDR in cancer. The phytochemicals may also be useful in eliciting food-drug interactions as a strategy to enhance the bioavailability and/or prolong the effects of P-gp substrate drugs.

## 4. Materials and Methods 

### 4.1. Materials

Doxorubicin, daunorubicin, capsaicin, [6]-gingerol, DMSO, PEG400 and formic acid were purchased from Sigma-Aldrich (St. Louis, MO, USA). Piperine and nonivamide were purchased from Tokyo Chemical Industry Co., Ltd. (Tokyo, Japan). HPLC-grade acetonitrile and distilled water were purchased from J.T. Baker (Philipsburg, NJ, USA).

### 4.2. Animal Study

The animal study was approved by the Ethics Committee for the Treatment of Laboratory Animals at Korea Animal Medical Science Institute (KAMSI IACUC 13-KE-014) and conducted following the standard operating procedures (SOPs).

Male ICR mice (age: 5–6 weeks old; body weight: 30 ± 2 g) were purchased from Samtako Co. (Osan, Gyeonggi-do, Korea), housed in plastic cages and given free access to a standard animal diet (Daejong, Seoul, Korea) and water. The environment was maintained under the following conditions: temperature, 23 ± 1 °C; 12-h light-dark cycle; and relative humidity, 50% ± 10%. The animals were acclimatized for a minimum period of 1 week and fasted overnight prior to experimentation.

The mice were randomly divided into four groups. Each group was pretreated with either control vehicle (a mixture of DMSO:PEG 400:distilled water; 5:45:50, *v*/*v*), piperine (10 mg/kg), capsaicin (5 mg/kg), or [6]-gingerol (5 mg/kg). The treatment solutions of piperine, capsaicin and [6]-gingerol were prepared by the dissolution of each compound in the DMSO:PEG 400:distilled water mixture at concentrations of 2, 1 and 1 mg/mL, respectively. The treatment solutions were administered intraperitoneally twice with τ = 60 min for control vehicle and piperine and τ = 30 min for capsaicin and [6]-gingerol. The LD50 values are 60 mg/kg for piperine (i.p.) [56], 7.6 mg/kg (i.p.) and 118.8 mg/kg (p.o.) for capsaicin [57,58] and 58.1 mg/kg for [6]-gingerol (i.p.) [59] in mice. The physiological conditions of the animals were carefully monitored during the study.

After 15 min of the second intraperitoneal administration, doxorubicin was administered via penile vein injection to all groups. For administration, doxorubicin was dissolved in distilled water at a concentration of 0.2 mg/mL and injected at a dose of 1 mg/kg. The volume of injected dosing vehicle was 5 mL/kg. Blood samples were collected by orbital bleeding 10 min after administration of the P-gp inhibitor (or control) and 5, 10, 15, 30 min and 2, 8, 12 and 24 h (80 μL each, *n* = 6 per each sampling time) after administration of doxorubicin. Plasma was harvested by centrifugation at 16,000× *g* for 10 min and immediately stored at −20 °C until analysis. In the tissue distribution study, the animals were anesthetized with diethyl ether and the kidney, brain, liver, testis, lung and heart were excised at 2, 8 and 24 h after intravenous injection of doxorubicin (*n* = 6 per sampling time). All excised tissues were accurately weighed and homogenized (Tissue Tearor, Biospec Products Inc., Bartlesville, OK, USA) with appropriate volumes of isotonic saline. The obtained tissue homogenates were stored at −20 °C until analysis.

### 4.3. LC-MS/MS

The levels of doxorubicin, piperine, capsaicin and [6]-gingerol in mice plasma and tissue samples were simultaneously determined by a validated LC-MS/MS assay. Daunorubicin and nonivamide were used as the internal standards for doxorubicin and the P-gp inhibitors, respectively. Sample analyses were conducted on an Agilent 6430 triple quadrupole mass spectrometer coupled with an Agilent 1200 HPLC (Agilent, Santa Clara, CA, USA). Good separation of the compounds was achieved on a Zorbax SB-C18 analytical column (2.0 × 150 mm, 3 μm, Agilent, Santa Clara, CA, USA) with SecurityGuard Cartridge Kit (Phenomenex, Torrance, CA, USA). Chromatographic separation was performed using a binary gradient mobile phase composed of acetonitrile (A) and 0.05% formic acid (B) and programmed as follows: initial conditions, 20% A (0.3 mL/min) for 0.1 min and 80% A for 3 min; increased to 90% A in 0.1 min and maintained for 1 min; returned to the initial composition; followed by a 3.2-min equilibration step (1.5 min for 0.3 mL/min and then 1.7 min for 0.5 mL/min). The ESI source was operated in positive mode with curtain and nebulizer gas set at 10 and 20 psi, respectively. The capillary voltage and gas temperature were 4000 V and 300 °C, respectively. The precursor/product ion pairs were monitored at *m*/*z* 544.2 → 397.1 for doxorubicin, 528.2 → 321.1 for daunorubicin, 286.2 → 201.0 for piperine, 306.2 → 137.0 for capsaicin, 277.2 → 177.1 for [6]-gingerol and 294.2 → 137.0 for nonivamide.

This assay utilized a simple protein precipitation in acetonitrile and achieved a lower limit of quantification (LLOQ) of 1 ng/mL for plasma and 2 ng/mL for tissue samples in sample volumes of 20 and 50 μL, respectively. The IS working solution (20 μL of 100 ng/mL IS solution for plasma and 50 μL of 100 ng/mL IS solution for tissue samples) and acetonitrile (50 μL for plasma and 80 μL for tissue samples) were added to 20 μL of plasma and 50 μL of tissue samples and mixed on a vortex mixer for 1 min. The mixture was then centrifuged at 16,000× *g* (Biofuge Fresco, Kendro, Osterode, Germany) for 10 min. For tissue samples, 100 μL of the supernatant was re-precipitated with an equal volume of acetonitrile and centrifuged at 16,000× *g* for 10 min. Finally, 50 μL distilled water was added to 50 μL of each supernatant, followed by mixing and injection of 10 μL of the sample mixture onto the LC-MS/MS. Data acquisition was performed with MassHunter Quantitative Analysis (Agilent, Santa Clara, CA, USA).

### 4.4. Data Analysis

The pharmacokinetic parameters were determined by non-compartmental analysis using Phoenix^®^ WinNonlin^®^ 6.4 (Certara, L.P., Princeton, NJ, USA). As the procedure entailed destructive sampling, the non-compartmental analysis was performed by using the average plasma concentration vs. time data. These parameters included terminal half-life (t_1/2_), area under the plasma concentration-time curve from time zero to infinity (AUC_inf_), apparent volume of distribution during the terminal phase (V_z_), the volume of distribution at steady state (V_ss_) and systemic clearance (CL). The peak serum concentration (C_max_) and the time to reach C_max_ (T_max_) were obtained directly from the observations. The tissue to plasma partition coefficients (K_p_) were calculated by dividing the doxorubicin concentration in each tissue by the doxorubicin concentration in the plasma.

### 4.5. Statistical Analysis

The means of the pharmacokinetic parameters and concentrations in the plasma and tissue were compared via unpaired *t*-test for unpaired data. A one-way ANOVA followed by Tukey’s post hoc test was applied to compare means across more than two groups. Values of *p* < 0.05 were considered statistically significant. Statistical analyses were conducted using SPSS (version 24.0, IBM Co., Armonk, NY, USA).

## Figures and Tables

**Table 1:** Effects of phytochemicals on the P-gp mediated transport in vitro.

Phytochemical	Cell Line	Substrate	Effects	Reference
Piperine	Caco-2	Digoxin Cyclosporin A	Inhibited P-gp mediated efflux transport	[4]
	Caco-2 L-MDR1	[^3^H] Digoxin	Inhibited P-gp mediated efflux transport	[5]
	MCF-7/DOX	Rhodamine 123 Mitoxantrone	Inhibited efflux activity of P-gp, MRP1 and BCRP	[30]
	A-549/DDP	Doxorubicin	Inhibited efflux activity of P-gp, MRP1 and BCRP	[30]
	MCF-7/DOX A-549/DDP	Doxorubicin	Increased cytotoxicity by reversing transporter mediated doxorubicin and mitoxantrone resistance	[30]
	MCF-7/DOX	Mitoxantrone	Increased cytotoxicity by reversing transporter mediated doxorubicin and mitoxantrone resistance	[30]
	MCF-7/DOX A-549/DDP	-	Inhibited transcription of the corresponding ABC transporter genes	[30]
Capsaicin	KB-C2	Daunorubicin	Increased cellular accumulation	[7]
	KB-C2	Rhodamine 123	Inhibited efflux transport	[7]
	KB-C2	Vinblastine	Increased vinblastine cytotoxicity by inhibition of efflux transporter	[7]
	Caco-2	[^3^H] Digoxin	Inhibited P-gp mediated efflux transport	[6]
[6]-Gingerol	KB-C2	Daunorubicin	Increased cellular accumulation	[7]
	KB-C2	Rhodamine 123	Inhibited efflux transport	[7]
	KB-C2	Vinblastine	Increased vinblastine cytotoxicity by inhibition of efflux transporter	[7]
	Caco-2 L-MDR1	[^3^H] Digoxin	Inhibited P-gp mediated efflux transport	[8]

**Table 2:** Average pharmacokinetic parameters of phytochemical P-gp inhibitors after intraperitoneal injection in mice.

Parameters	Piperine (10 mg/kg)	Capsaicin (5 mg/kg)	[6]-Gingerol (5 mg/kg)
t_1/2_ (h)	6.30	0.33	1.36
T_max_ (h)	0.75	0.17	0.17
C_max_ (ng/mL)	5050.97	118.90	153.37
AUC_inf_ (ng·h/mL)	9956.92	66.83	66.34
CL/F (mL/min/kg)	16.74	1247.02	1256.20
V_z_/F (L/kg)	9.12	35.66	147.54

t_1/2_, terminal half-life; T_max_, time to reach C_max_; C_max_, peak plasma concentration; AUC_inf_, area under the plasma concentration-time curve from time zero to infinity; CL/F, systemic clearance; V_z_/F, apparent volume of distribution during the terminal phase.

**Table 3:** Average pharmacokinetic parameters of doxorubicin after intravenous injection at 1 mg/kg in mice pretreated with phytochemical P-gp modulators.

Parameters	Pretreatment			
	Control	Piperine	Capsaicin	[6]-Gingerol
t_1/2_ (h)	17.39	15.68	13.27	15.48
C_0_ (ng/mL)	3984.48	3404.57	4428.01	4036.96
AUC_inf_ (ng·h/mL)	537.08	651.03	661.02	579.44
CL (mL/min/kg)	31.03	25.60	25.21	28.76
V_z_ (L/kg)	46.72	34.75	28.96	38.53

t_1/2_, terminal half-life; C_0_, initial plasma concentration; AUC_inf_, area under the plasma concentration-time curve from time zero to infinity; CL, systemic clearance; V_z_, apparent volume of distribution during the terminal phase.

**Table 4:** Doxorubicin tissue concentrations and tissue to plasma partition coefficients (K_p_) in mice pretreated with piperine, capsaicin and [6]-gingerol 2, 8 and 24 h after intravenous injection of doxorubicin at 1 mg/kg (*n* = 6, mean ± SD).

		Control		Piperine		Capsaicin		[6]-Gingerol	
		Concentration (ng/mL or ng/g)	K_p_	Concentration (ng/mL or ng/g)	K_p_	Concentration (ng/mL or ng/g)	K_p_	Concentration (ng/mL or ng/g)	K_p_
2 h	Plasma	19.4 ± 5.0	-	18.2 ± 8.1	-	18.5 ± 4.8	-	23.4 ± 5.3	-
	Kidney	2303 ± 686.9	89.6 ± 24.3	2840.8 ± 431.2	199.8 ± 76.3 *	3131.9 ± 255.6 *	174.5 ± 39.9 *	2756.7 ± 536.7	125.8 ± 36.2
	Brain	8.2 ± 2.4	0.3 ± 0.1	7.0 ± 3.5	0.5 ± 0.3	18.1 ± 7.7 *	1.0 ± 0.3 *	7.9 ± 4.2	0.4 ± 0.3
	Liver	909.7 ± 458.5	35.4 ± 17.0	2488.1 ± 1120.7	192.5 ± 137.1 *	4106.1 ± 2248 *	220 ± 92.6 *	1450.1 ± 924.9	64.5 ± 41.9
	Testis	25.2 ± 8.2	1.0 ± 0.2	11.9 ± 5.1	0.9 ± 0.6	28.1 ± 6.7	1.5 ± 0.3	30.3 ± 12.8	1.4 ± 0.6
	Lung	2356.3 ± 1647.2	85.9 ± 51.5	932.8 ± 350.6	78.6 ± 46.2	1916.4 ± 386.2	105.3 ± 24.2	2247.3 ± 620.8	102.5 ± 37.9
	Heart	1302.9 ± 246.6	51.9 ± 13.9	1097.4 ± 246.3	77.0 ± 31.3	815.4 ± 138.4 *	45.3 ± 12.5	1021.1 ± 261.8	47.5 ± 19.0
8 h	Plasma	8.4 ± 2.0	-	12.4 ± 2.1 *	-	12.3 ± 2.6 *	-	9.9 ± 3.2	-
	Kidney	673.5 ± 104.5	87.2 ± 20.4	811.4 ± 168.8	72.9 ± 9.3	777.9 ± 210.1	74.0 ± 16.4	694.6 ± 188.8	93.9 ± 27.3
	Brain	-	-	-	-	-	-	-	-
	Liver	891.1 ± 276.6	118.6 ± 52.7	2413.8 ± 690.6 *	218.0 ± 63.9 *	1357.8 ± 287	137.7 ± 57.0	999 ± 234.5	137.0 ± 43.5
	Testis	15.4 ± 10.9	2.0 ± 1.2	10.0 ± 1.7	0.9 ± 0.2	19.9 ± 18.4	1.9 ± 1.5	19.3 ± 8.7	2.6 ± 1.1
	Lung	851.2 ± 164	108.4 ± 21.2	821 ± 163.7	76.6 ± 25.9	888.6 ± 218.3	86.1 ± 27.0	779.7 ± 184.7	105.2 ± 28.2
	Heart	531.7 ± 315.6	63.8 ± 20.6	572.4 ± 133.6	51.7 ± 10.4	544.6 ± 65.2	55.0 ± 19.3	577.2 ± 132	77.5 ± 16.4
24 h	Plasma	4.6 ± 0.8	-	6.1 ± 1.5	-	5.6 ± 2.1	-	4.9 ± 0.6	-
	Kidney	425.6 ± 78.7	90.3 ± 20.9	394.4 ± 26.5	67.8 ± 17.9	424 ± 57	82.6 ± 30.4	442.2 ± 47.4	91.6 ± 8.0
	Brain	-	-	-	-	-	-	-	-
	Liver	364.5 ± 58.1	76.6 ± 13.6	524.1 ± 118.7	91.0 ± 30.5	362.3 ± 208.4	63.1 ± 16.7	334.4 ± 94.6	68.7 ± 17.9
	Testis	17.8 ± 3.8	3.8 ± 1.1	14.8 ± 2.3	2.6 ± 0.9	17.5 ± 3.1	3.4 ± 1.2	23.4 ± 5.4	4.9 ± 1.3
	Lung	546.6 ± 128.6	116.0 ± 33.7	485.5 ± 141.8	84.7 ± 34.2	647.4 ± 362.1	122.7 ± 76.6	499.2 ± 152	101.3 ± 18.6
	Heart	218.2 ± 43.8	46.7 ± 13.6	190.8 ± 46.8	33.3 ± 13.5	178.2 ± 32.3	33.9 ± 9.4	253.4 ± 66.2	51.5 ± 7.3

* *p* < 0.05 vs. control.
